# Supplementary material for: NMR-Relaxometric Investigation of Mn(II)-Doped Polyoxometalates in Aqueous Solutions
Source: Int J Mol Sci. 2023 Apr 15;24(8):7308. doi: 10.3390/ijms24087308 (PMC10139238; doi:10.3390/ijms24087308)
Supplement: Supplementary file 1 [file ijms-24-07308-s001.zip › ijms-2335501-supplementary.pdf]

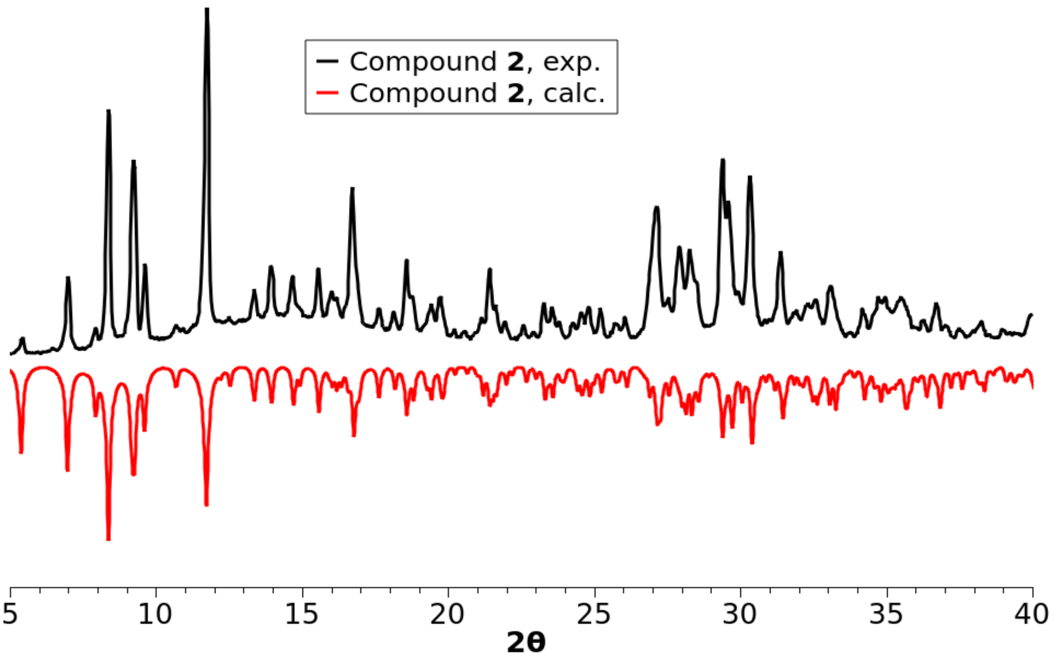

Figure S1. X-ray powder diffraction data for **2**.

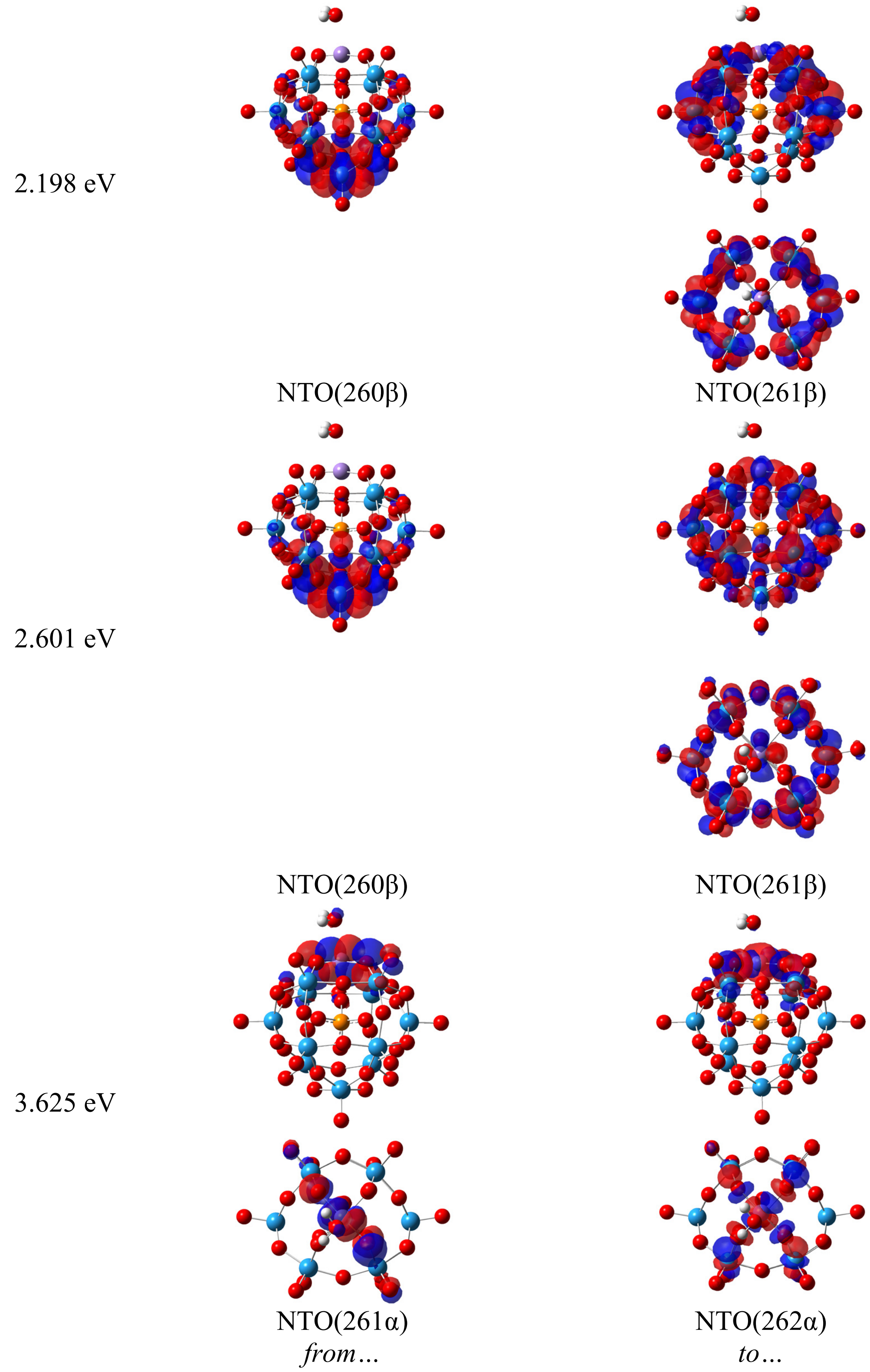

Figure S2. NTO analysis for $[(Mn^{II}(H_2O))PW_{11}O_{39}]^{5-}$. Color codes: Mn – purple, W – cyan, O – red, P – orange, H – white.

Table S1. Atomic ratios W/Mn for $[(Mn(H_2O))PW_{11}O_{39}]^{5-}$ peak calculated from HPLC-ICP-AES-data.

W/Mn	Mn 259.3 nm	Mn 260.5 nm	Mn 279.4 nm
W 209.8 nm	10.9±0.6	11.5±0.6	11.1±0.5
W 229.4 nm	11.3±0.5	11.2±0.5	11.5±0.6
W 239.7 nm	10.8±0.4	11.0±0.4	11.0±0.4

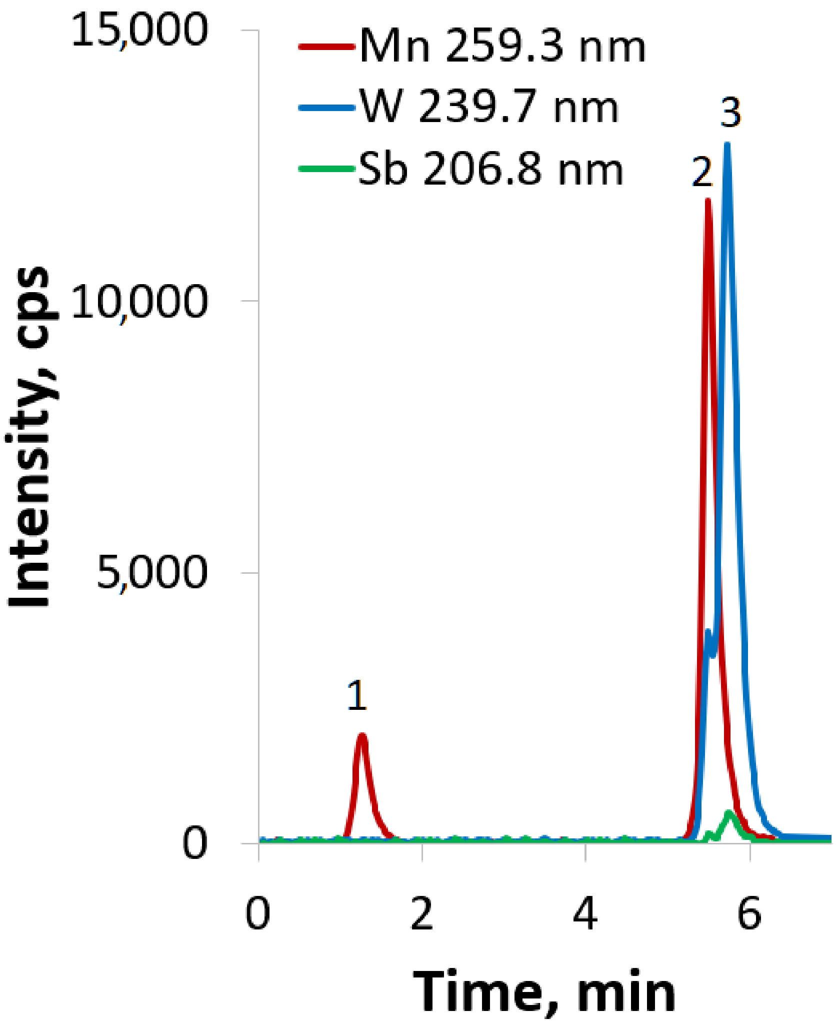

Figure S3. HPLC-ICP-AES chromatogram of **2** in water at natural pH scaled from the start of the elution.

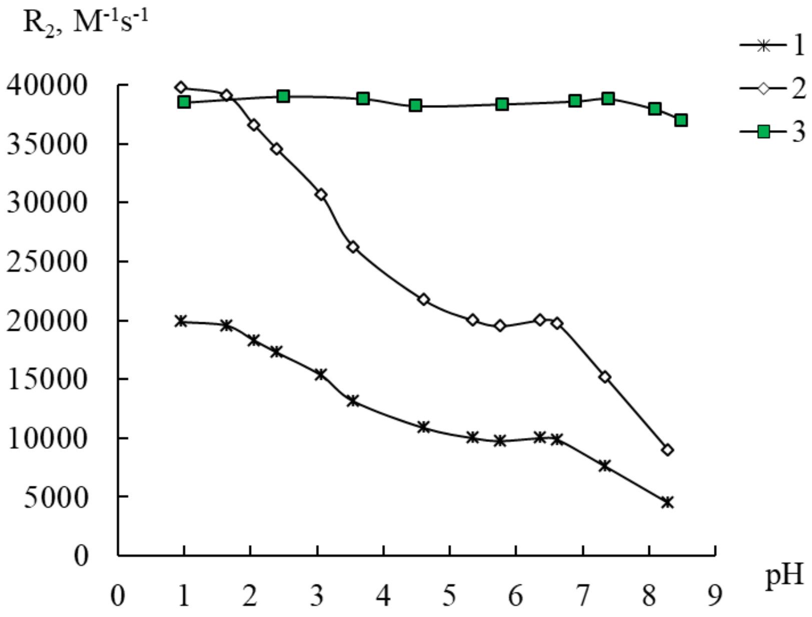

Figure S4. Dependence of the spin-spin relaxivity R_2 on pH in a 0.1 M solution of compound **3** in water. Calculations of relaxivity were carried out taking into account manganese(II) concentrations of 0.4 mM (1) and 0.2 mM (2). For comparison, data are presented for a $MnCl_2$ solution with a manganese(II) aqua ion content of 0.4 mM (3).

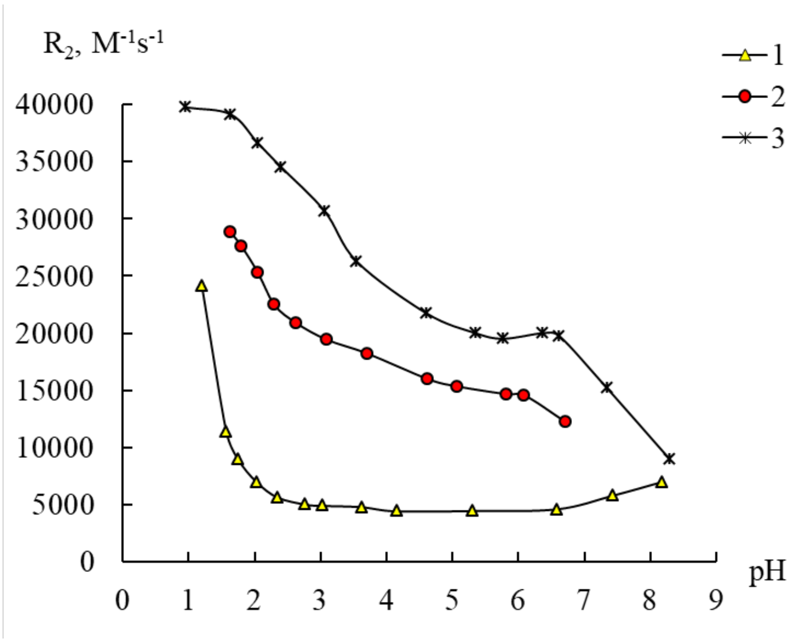

Figure S5. Changes in the relaxation efficiency R2 (a) on the pH of solutions of compounds **1** (0.4 mM) (1), 2 (0.133 mM) (2), and 3 (0.1 mM) (3). CMn(II) 0.2 (3), 0.4 mM (1, 2).

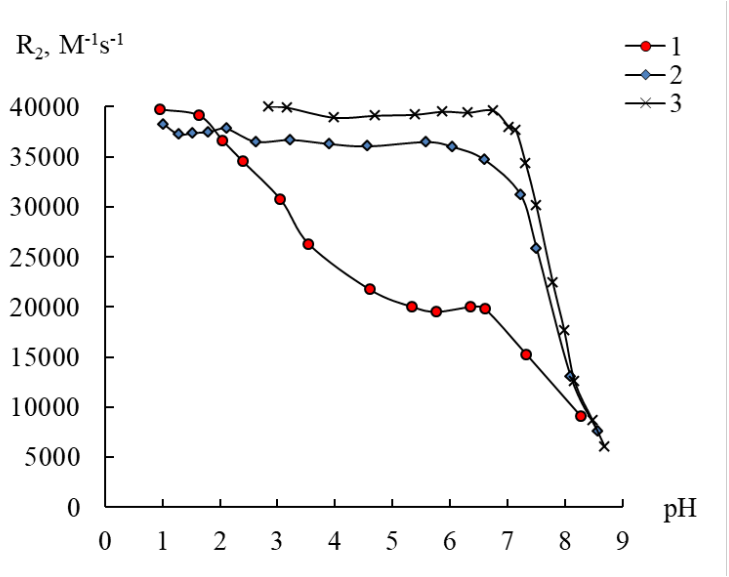

Figure S6. Changes in the relaxation efficiency R2 on pH 0.1 mM solutions of compound **3** in water (1) and in PEI solution (2), and Mn(II) ions in PEI solution (3). $C_{Mn(II)}$ 0.2 (1, 2), 0.4 mM (3), C_{PEI} 10 mM (2, 3).

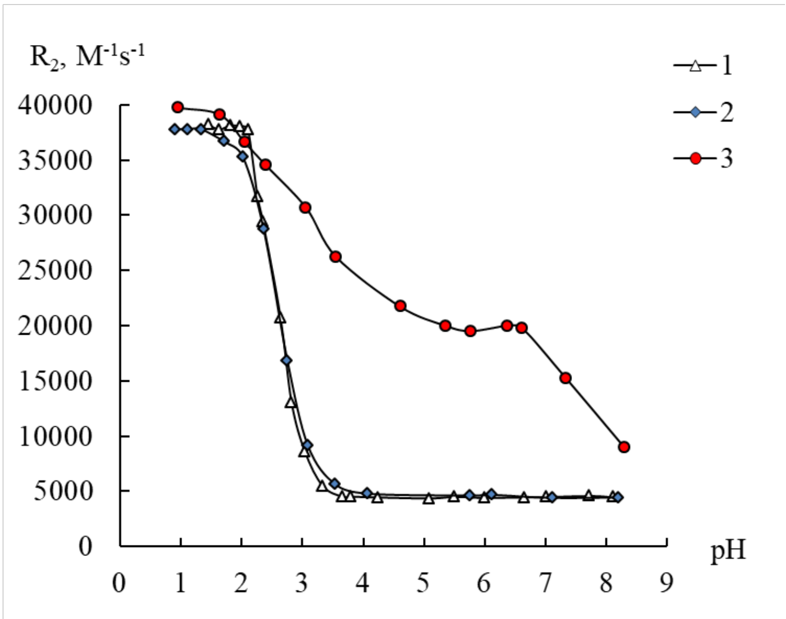

Figure S7. Changes in relaxation efficiency R_2 on pH of Mn(II)-EDTA systems (1), compound **1** – EDTA (2), aqueous solution of **1** (3). $C_{Mn(II)}$ 0.2 (2), 0.4 mM (1, 3), C_{EDTA} 1 mM (1, 2).

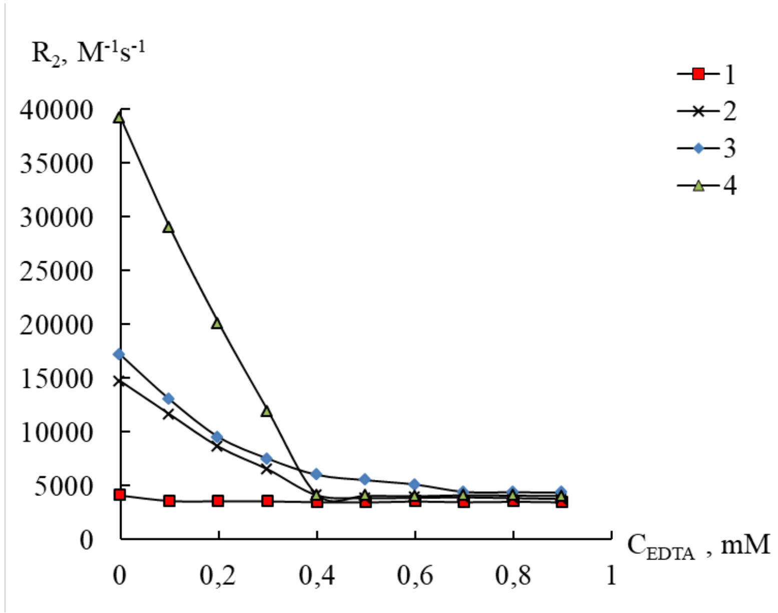

Figure S8. Change in the relaxation efficiency R_2 on the content of EDTA in solutions of compounds **1-3** (1-3) and in an aqueous solution of aqua ions Mn(II) (4), $C_{Mn(II)}$ 0.2 (3), 0.4 mM (1, 2, 4), pH 5.5.

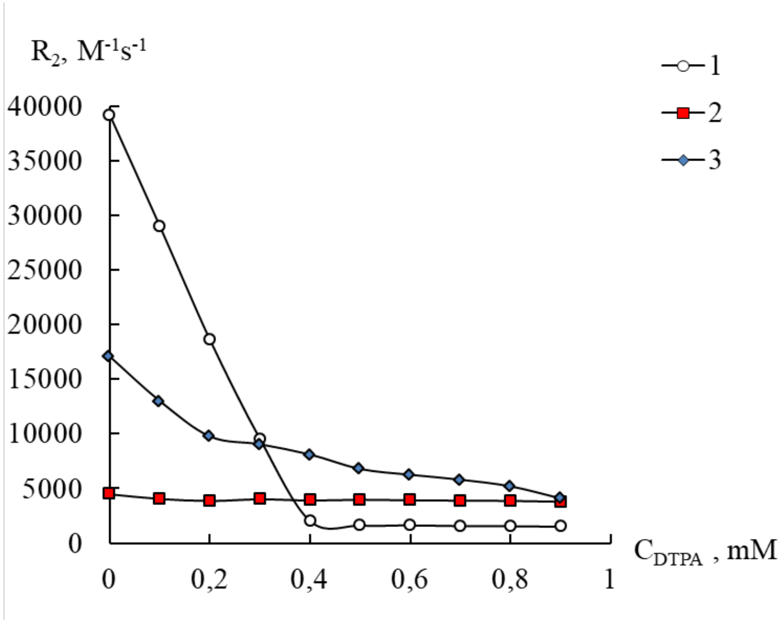

Figure S9. Dependence of the relaxation efficiency R_2 on the concentration of DTPA in aqueous solutions of Mn(II) (1) and compounds **1** (2) and **3** (3). $C_{Mn(II)}$ 0.2 (3), 0.4 mM (1, 2), pH 5.5.

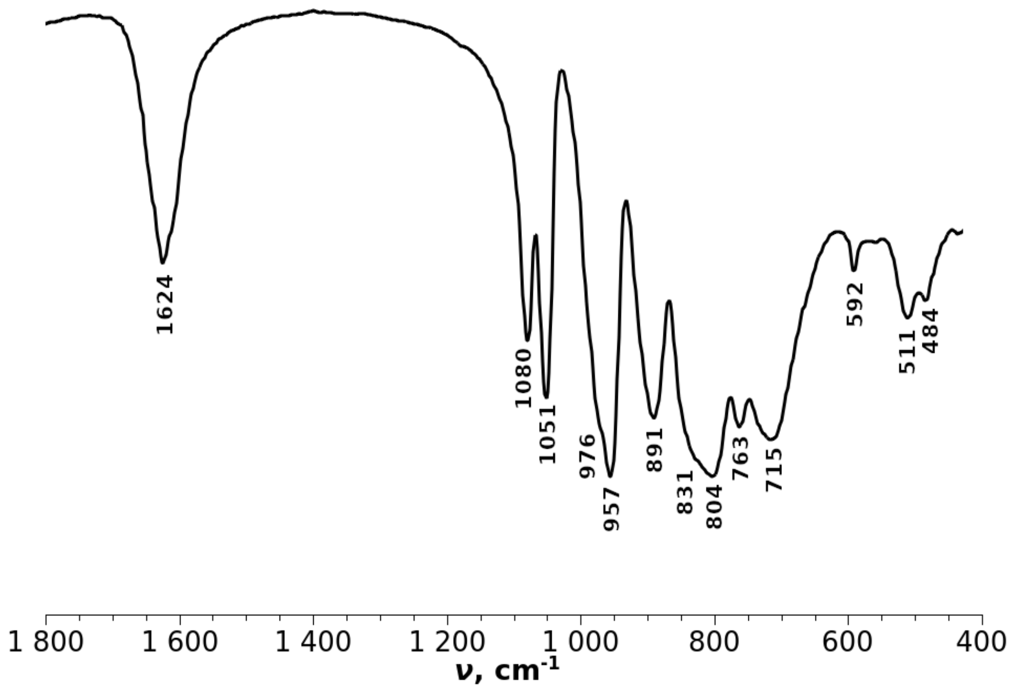

Figure S10. IR spectrum of complex **1**.

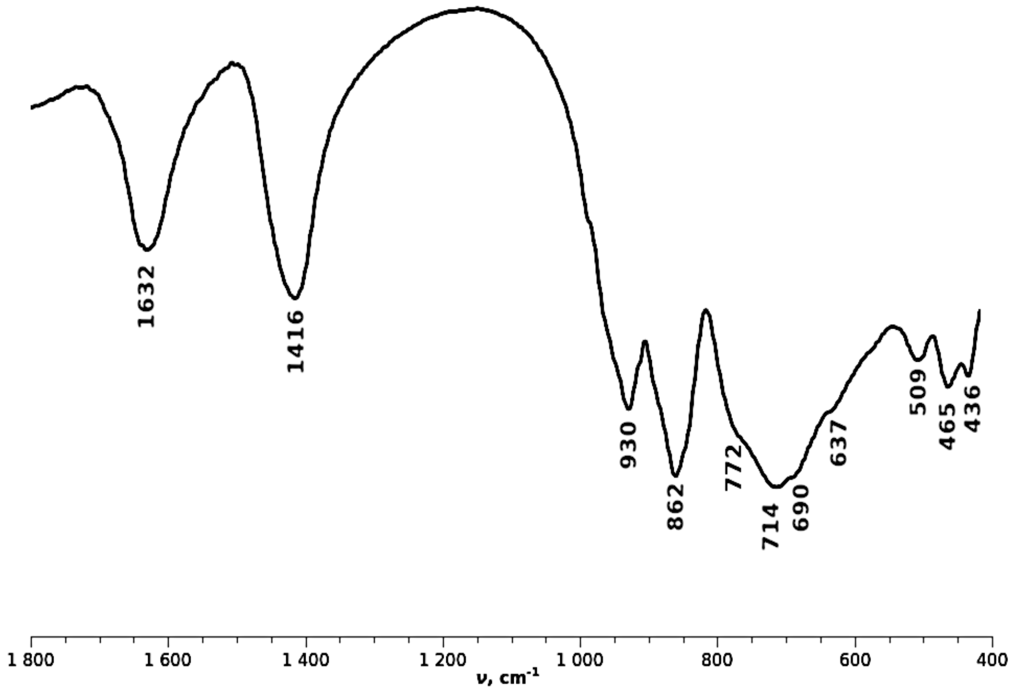

Figure S11. IR spectrum of complex **2**.

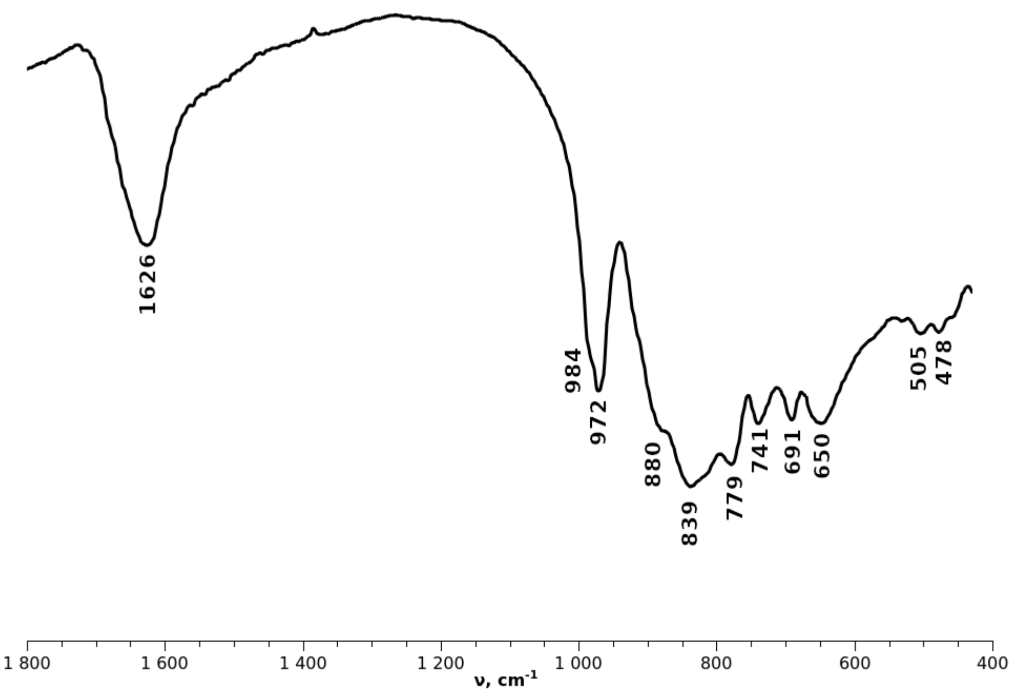

Figure S12. IR spectrum of complex 3.
